# Virucidal activity of three standard chemical disinfectants against Ebola virus suspended in tripartite soil and whole blood

**DOI:** 10.1038/s41598-023-42376-8

**Published:** 2023-09-21

**Authors:** Hulda R. Jonsdottir, Daniel Zysset, Nicole Lenz, Denise Siegrist, Yelena Ruedin, Sarah Ryter, Roland Züst, Yannick Geissmann, Rahel Ackermann-Gäumann, Olivier B. Engler, Benjamin Weber

**Affiliations:** 1 Spiez Laboratory, Federal Office for Civil Protection, Spiez, Switzerland; 2 Department of Rheumatology, Immunology, and Allergology, Inselspital University Hospital, Bern, Switzerland; 3 Department of BioMedical Research, University of Bern, Bern, Switzerland; 4 Institute of Microbiology, Lausanne University Hospital, University of Lausanne, 1011 Lausanne, Switzerland; 5 Present Address: Agroscope, Federal Office for Agriculture, Bern, Switzerland; 6 Present Address: Bernhard Nocht Institute for Tropical Medicine, Hamburg, Germany; 7 Present Address: ADMED Microbiologie, La Chaux-de-Fonds, Switzerland

**Keywords:** Virology, Ebola virus

## Abstract

Proper disinfection and inactivation of highly pathogenic viruses is an essential component of public health and prevention. Depending on environment, surfaces, and type of contaminant, various methods of disinfection must be both efficient and available. To test both established and novel chemical disinfectants against risk group 4 viruses in our maximum containment facility, we developed a standardized protocol and assessed the chemical inactivation of the two Ebola virus variants Mayinga and Makona suspended in two different biological soil loads. Standard chemical disinfectants ethanol and sodium hypochlorite completely inactivate both Ebola variants after 30 s in suspension at 70% and 0.5% v/v, respectively, concentrations recommended for disinfection by the World Health Organization. Additionally, peracetic acid is also inactivating at 0.2% v/v under the same conditions. Continued vigilance and optimization of current disinfection protocols is extremely important due to the continuous presence of Ebola virus on the African continent and increased zoonotic spillover of novel viral pathogens. Furthermore, to facilitate general pandemic preparedness, the establishment and sharing of standardized protocols is very important as it allows for rapid testing and evaluation of novel pathogens and chemical disinfectants.

## Introduction

Zaire Ebola virus (EBOV) is a zoonotic filovirus that causes severe hemorrhagic fever in humans and can present with a case fatality rate (CFR) of up to 90%^1^. The West African Ebola virus disease (EVD) outbreak of 2014–16 has raised concerns about safe and efficacious disinfection of treatment centers, hospital rooms, personal protective equipment, patient transfer vehicles, and airplanes carrying infected persons. Successful disinfection of such a complex mixture of different materials, e.g., fabrics, mattresses, plastics, rubbers, and metals, is extremely challenging. Furthermore, the diverse composition of infectious bodily fluids, the primary transmission route of EBOV complicates disinfection procedures, and negatively affects chemical inactivation of infectious virus^2^. The World Health Organization (WHO) recommends using alcohol-based hand rubs, soap and running water, laundry detergents, and sodium hypochlorite (NaOCl) for chemical inactivation in EVD treatment centers (WHO Interim Infection Prevention, 2014). Cook et al. evaluated the virucidal activity of 70% ethanol and 0.5% NaOCl on steel carriers and both formulations inactivated EBOV in the recommended time. Intriguingly, they discovered an outbreak variant dependency where the Makona variant from the West African outbreak of 2014–2016 was less susceptible to weaker concentrations of NaOCl and inactivation in 70% ethanol took longer than the Central African variants Kikwit and Mayinga (the prototype strain obtained during the first recorded EVD outbreak in 1976)^3^. Although disinfection of EBOV with NaOCl is standard operating procedure, peracetic acid (PAA) is increasingly preferred over NaOCl for certain disinfection purposes, e.g., wastewater treatment^4^, and it is also commonly used in fogging systems to decontaminate hospitals^5^, schoolrooms^6^, and public transport vehicles^7^. Liquid PAA has also been used to decontaminate personal protective equipment (PPE) during hospital treatment of a laboratory worker exposed to EBOV in Hamburg, Germany^8^. Storage conditions for PAA and NaOCl stock solutions are highly similar, temperature should range between 15 and 25 °C, stocks should be protected from sunlight, and storage space must be well ventilated. While NaOCl is not combustible PAA is flammable both as liquid and vapor. On the other hand, PAA is relatively unaffected by organic loads and temperature, providing an advantage for the disinfection of contaminated biological materials. Additionally, PAA is a balanced solution consisting of peracetic acid, acetic acid, and hydrogen peroxide and once broken down it is more environmentally friendly^9,10^ than NaOCl, which can lead to production of trihalomethanes and other carcinogenic halo-organic compounds^11^. To evaluate PAA as a possible alternative for chemical inactivation of EBOV, we compared the efficacy of three different chemical disinfectants, ethanol, NaOCl, and PAA against EBOV variants Mayinga and Makona. We developed a standardized testing protocol based on DIN EN 14,476:2013 + A1:2015 and the guideline developed by the Robert Koch Institute in Germany^12^ using different organic soil loads. The established protocol allows for evaluation of the chemical inactivation of different disinfectants using the same workflow, facilitating reproducibility, and minimizing experimental variation. In the current study, we observed complete inactivation of the EBOV variants Mayinga and Makona, in suspension after 30 s incubation using recommended concentrations of ethanol, NaOCl, and PAA.

## Materials and methods

### Cell culture

Cells from African Green Monkey kidney (Vero E6) were acquired from the American Type Culture Collection (ATTC CRL-1586) and maintained in Minimum Essential Medium (MEM) supplemented with 10% fetal bovine serum (FBS), 2 mM L-Glutamine, 1 × non-essential amino acids (NEAA), 0.1 U/ml penicillin, and 0.1 mg/ml streptomycin. All cell culture media and reagents were produced by Seraglob and acquired from Bioswisstec, Schaffhausen Switzerland.

### Virus propagation and concentration

Two separate strains of Zaire Ebola virus (EBOV), the type species Mayinga variant (Mayinga-76) as well as the 2014–16 West Africa outbreak strain, Makona (Gueckedou-C05), were propagated for 5 days on Vero E6 cells in 30 ml of MEM supplemented with 2% FBS (2% MEM) in 150 cm^2^ flasks (Corning International, Fisher Scientific, Reinach, Switzerland) and concentrated with 100 kD Amicon Ultra-15 centrifugal filters (Merck Millipore, Merck & Cie, Schaffhausen, Switzerland). Briefly, once harvested, 15 ml of virus supernatant was centrifugated at 3000 g for 20 min at 4 °C and concentrated virus harvested by flushing the filters with 750 µl of 2% MEM. Each column was used twice (total input volume 30 ml) and the volume of the concentrate (total output volume 1.5 ml) diluted tenfold before aliquoting, resulting in 15 ml of concentrated high titer virus stock (10^7^–10^8^ FFU/ml) from each flask.

### Water of standardized hardness (WSH)

To ensure experimental reproducibility, water of standardized hardness (WSH) was prepared as previously described^13^. Briefly, 2.1 g MgCl_2_ and 2.3 g CaCl_2_ were diluted in 50 ml of ddH_2_O and designated as solution A. 1.75 g NaHCO_3_ was diluted in 50 ml of ddH_2_O and designated as solution B. All salts were sourced from Merck Millipore via Grogg Chemie AG, Stettlen, Switzerland. Both solutions were sterile filtered through a 0.22 µm syringe filter (Sartoris miniSART, Fischer Scientific, Reinach, Switzerland) prior to use. Neither solution was used after their expiration date, 30 and 7 days for solutions A and B, respectively. For 50 ml WSH, 300 µl solution A and 400 µl solution B were diluted in 49.3 ml ddH_2_O, pH set to 7 ± 0.2, and sterile filtered through a 0.22 µm syringe filter. After preparation, WSH was used within 16 h.

### Decontamination solutions and soil loads

Ethanol, absolute for analysis (Merck Millipore, Grogg Chemie AG, Stettlen, Switzerland) was diluted to 87.5% v/v, 35% sodium hypochlorite (NaOCl, Honeywell Fluka, Fischer Scientific, Reinach Switzerland) was diluted to 0.625% v/v, and 40% peracetic acid (PAA, Sigma Aldrich, Buchs, Switzerland) was diluted to 0.25% v/v in 800 µl WSH. Virucidal activity of these decontamination solutions was assessed in two different biological soil types. Tripartite soil, intended as a substitute for mucosal secretions, containing 100 µl 5% bovine serum albumin (BSA), 400 µl 0.4% mucin, and 140 µl 5% tryptone (all sourced from Sigma Aldrich, Buchs, Switzerland) representing a medium soil load as previously described^13–15^. For a heavier soil load, whole blood was drawn with informed consent from a healthy 24-year-old donor never exposed to Ebola virus using BD Vacutainer K2E (EDTA) Plus Blood Collection Tubes (BD Switzerland Sàrl, Eysins, Switzerland) and used within 72 h. 1 ml concentrated Ebola virus was added to 640 µl of each soil load.

### Virucidal activity of decontamination solutions

The virucidal activity of disinfectants was assessed by adding 200 µl virus/soil mixture to 800 µl of the diluted disinfectants mentioned above achieving 70 and 40% final concentrations for ethanol, 0.5 and 0.05% for NaOCl, and 0.2 and 0.02% for PAA, and incubating for 30 s. Subsequently, 50 µl of the virucidal test was inoculated directly into 25 cm^2^ flasks to determine residual infectivity after treatment. Another 50 µl were added to 450 µl of cell culture media (to dilute ethanol to a non-working concentration) or cell culture media supplemented with neutralizer (1% sodium thiosulfate to neutralize NaOCl) or cell culture media supplemented with both neutralizer (0.25% sodium thiosulfate) and additional 25 mM of HEPES (to neutralize and buffer PAA; Gibco, Fischer Scientific, Reinach, Switzerland) and diluted tenfold in a deep well plate (Eppendorf, Schönenbuch, Switzerland). 150 µl of each dilution was then inoculated on Vero E6 cells (median passage number 42) in a 24-well plate (Techno Plastic Products, TPP, Trasadingen, Switzerland) and incubated at RT for 1 h. After incubation, 1 ml of overlay media, 2% MEM + 1% methylcellulose 90 H G 4000 cP (Sigma Aldrich, Buchs, Switzerland), was added on top and cells incubated at 37 °C without CO_2_ for 7 days.

### Controls

#### Negative control

Two negative controls were applied both in 24-well format and 25 cm^2^ flasks during each experiment, a cell control treated with cell culture media alone to determine general cell health and a toxicity control where cells were treated with the same concentration of disinfectants used for virucidal tests to confirm that any reduction in viral titer is not due to cytotoxicity.

#### Interference control

To confirm that cell incubation with disinfectants does not interfere with their ability to replicate virus, tenfold dilutions of either disinfectant or PBS were incubated for 1 h and removed prior to addition of virus. Experiments were considered valid when infectious titers of interference controls determined by focus assay were within 0.5log from positive control. For PAA, additional interference control was set up in a 25 cm^2^ flask by incubating 50 µl of the disinfectant/soil mixture for 1 h at RT and adding 50 ul of the positive control to ensure sufficient cell health for virus replication.

#### After-effects control

To demonstrate the neutralization of viral inactivation by disinfectants, 200 µl virus/soil mixture was incubated in 800 µl of tenfold diluted disinfectant (ethanol) or tenfold diluted disinfectant mixed with sodium thiosulfate neutralizer, 1% for NaOCl and 0.25% for PAA, for 15 min at RT. Experiments were considered valid when infectious titers of after-effects controls determined by focus assay were within 0.5log from positive control.

#### Neutralizer control

To demonstrate the capacity of neutralizing virucidal activity with sodium thiosulfate without affecting virus infectivity, virus/soil mixture was titrated in media containing the same concentration of neutralizer as inactivation tests. Experiments were considered valid when infectious titers of neutralizer controls were within 0.5log from positive control. Applied only for NaOCl (1%) and PAA (0.25%).

#### Positive control

To estimate maximum viral replication without intervention, 200 µl virus/soil mixture was diluted in 800 µl WSH, titrated, and incubated in cell culture media alone. Experiments were considered valid when infectious titers of other controls were determined within 0.5log from the observed values for this control.

#### Complete-kill control

To determine residual infectivity, 50 µl of inactivation tests (same volume as used for titration in 24-well plates) were inoculated in 2 ml of cell culture media on Vero E6 cells in a 25 cm^2^ flask (Techno Plastic Products, TPP, Trasadingen, Switzerland) and incubated for 1 h at RT and subsequently supplemented with additional 10 ml of media. Virus growth estimated by presence of cytopathic effect (CPE) and quantification of viral RNA by qRT-PCR where ΔCt (d0 − d7) ≥ 3 was considered indicative of viral replication. All control values and the range of experimental validity (green) are summarized in Figs. S1 and S2 and Tables S1 and S2.

### Focus assay

Infectious titers were determined by a slightly modified version of a standardized focus assay for highly pathogenic viruses previously established in the laboratory^16^. Briefly, overlay medium, was removed and wells washed once with 1 ml PBS and then fixed in 1 ml 10% Roti^®^Histofix (Carl Roth, Karlsruhe, Germany) for 1 h at room temperature (RT) and then washed once with 0.5 ml PBS-T (0.05% Tween-20; Sigma Aldrich, Buchs, Switzerland, in PBS; Seraglob, Schaffhausen, Switzerland). Subsequently, the cell layer was permeabilized with 0.5 ml 0.1% Triton-X 100 (Sigma Aldrich, Buchs, Switzerland) in PBS for 5 min, followed by a washing step with PBS-T. The cell layer was then incubated overnight at 4 °C with 200 µl of rabbit polyclonal primary antibody against Ebola virus (subtype Zaire, strain Mayinga 1976) GP-RBD/Glycoprotein (Sino Biological, Pennsylvania, USA) diluted 1:2000 in 4% skim milk and 10% FBS in PBS-T. The following day, primary antibody was removed, and the cell layer washed again with PBS-T, prior to incubation with 200 µl HRP-goat-anti rabbit secondary antibody (BioConcept, Alschwil, Switzerland) diluted 1:1000 in PBS-T for 1 h at RT. For visualization, one tablet (20 mg) of 3-Amino-9-Ethylcarbazole was dissolved in 2.25 ml N,N-Dimethylformamide and then further diluted in 47.5 ml 0.05 M sodium acetate (Sigma Aldrich, Buchs, Switzerland). Immediately prior to staining, 0.05% H_2_O_2_ was added. Limit of detection (LOD) was 10 FFU/well.

### qRT-PCR

Viral RNA in supernatant from complete-kill controls was quantified using the TaqMan™ Fast-Virus-1 Step master mix according to the manufacturer’s instructions (Fisher Scientific, Reinach, Switzerland) with the following cycling parameters: 50 °C for 1 min, 95 °C for 20 s, 45 cycles of 95 °C for 3 s and 60 °C for 30 s using the LightCycler 96 system (Roche, Basel, Switzerland) using the following adapted primers^17^ targeting Zaire Ebola virus, Fwd: 5′-TTTTCAATCCTCAACCGTAAGGC2-3′, Rev: 5′-CAGTCCGGTCCCARAATRTG-3′, Probe: 5′-FAM-CATGTGCCRCCCCATCGCTGC-BHQ-1–3′.

## Results

### Chemical inactivation of EBOV

**Figure 1:**
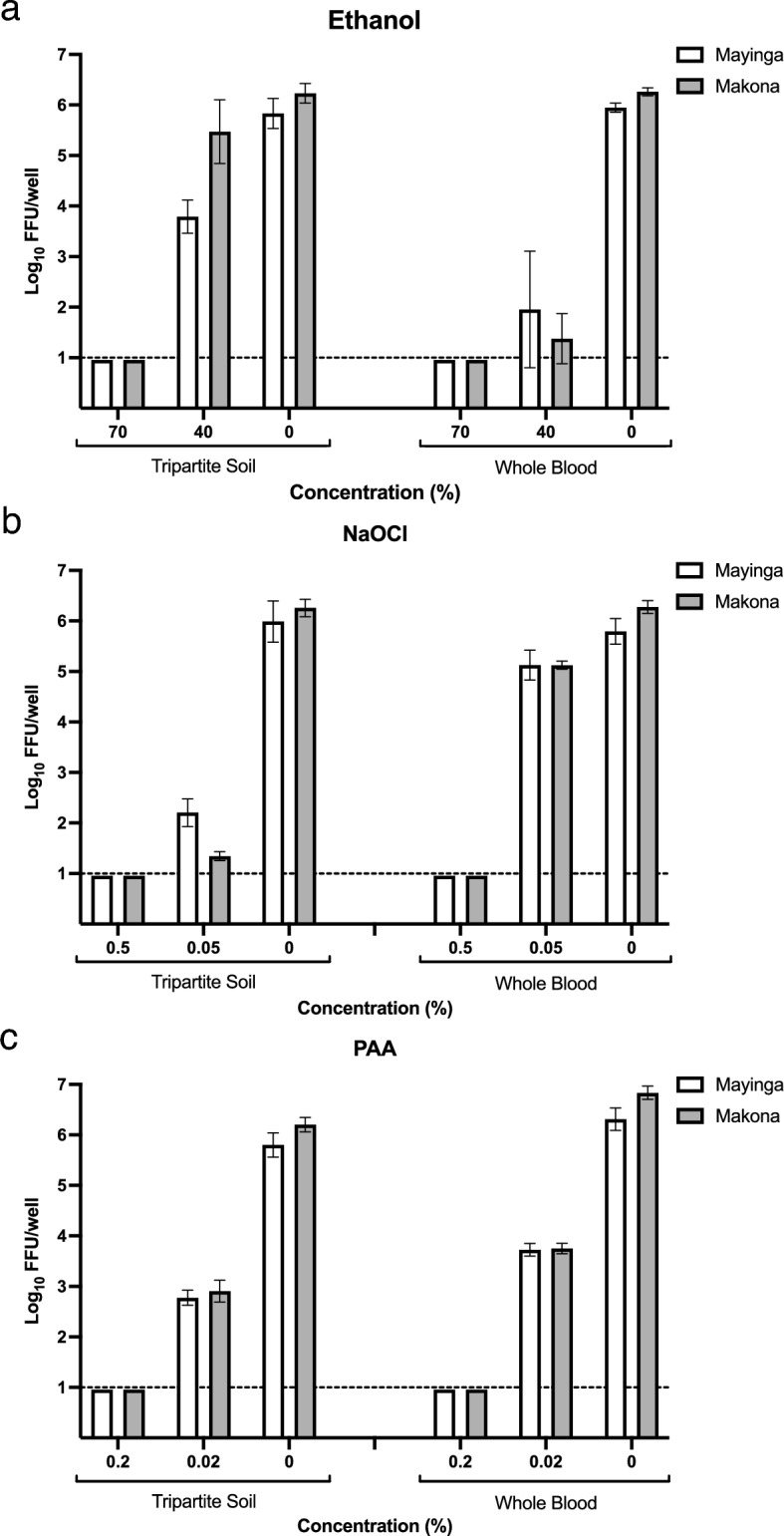
Residual infectivity of EBOV suspended in tripartite soil or whole donor blood after treatment with disinfectants in suspension for 30 s. EBOV Makona and Mayinga suspended in either tripartite soil or whole blood and treated with (**a**) 70% or 40% Ethanol, (**b**) 0.5% or 0.05% NaOCl, and (**c**) 0.2% or 0.02% PAA. Corresponding positive controls are indicated as 0%. Dashed line indicates LOD of 10 FFU/well. Bars indicate mean ± SD of 2 independent experiments with n = 6 for test concentrations and n = 4 for controls.

Treatment of the EBOV variants Mayinga and Makona suspended in tripartite soil with 70% ethanol for 30 s results in complete virus inactivation as assessed by focus assay (Fig. 1a). Furthermore, no virus replication was observed in complete-kill (CK) controls as assessed by either cytopathic effect (CPE, Fig. S2a) or qPCR analysis (ΔCt, Table S3), indicating complete viral inactivation. The same pattern of inactivation is observed when EBOV variants were inactivated in whole donor blood (Fig. 1a, Fig. S2a, and Table S3). In contrast, treatment with a lower concentration of ethanol (40%) is not enough to inactivate either variant in either soil type. However, a difference in susceptibility to this concentration is observed between the variants, with Makona being more resistant when suspended in tripartite soil presenting with residual infectious titer of 5.47 Log_10_ FFU/well while residual titer of 3.79 Log_10_ FFU/well was observed for Mayinga (Fig. 1a, Table 1). When applied to EBOV variants suspended in donor blood, treatment with 40% ethanol exhibits increased inactivation capacity with minimal difference in residual infectivity between the two variants, 1.95 and 1.38 Log_10_ FFU/well for Mayinga and Makona, respectively (Fig. 1a, Table 2). No remaining infectivity is observed for either Mayinga or Makona suspended in tripartite soil or donor blood after treatment with 0.5% NaOCl for 30 s as assessed by focus assay (Fig. 1b) and CK controls (Fig. S2b, Table S3). In contrast, treatment with 0.05% NaOCl results in residual infectivity of 2.2 Log_10_ FFU/well for Mayinga and 1.35 Log_10_ FFU/well for Makona when suspended in tripartite soil (Fig. 1b, Table 1). Interestingly, this concentration exhibited much less inactivation capacity in donor blood with residual virus infectivity over 5 Log_10_ FFU/well for both variants (Fig. 1b, Table 2). Peracetic acid exhibits the same inactivation pattern for both Mayinga and Makona as observed for the other disinfectants. 0.2% PAA results in complete inactivation of the variants in both tripartite soil and whole blood (Fig. 1c, Fig. S2c, Table S3) while 0.02% results in residual infectivity of 2.78 Log_10_ FFU/well for Mayinga and 2.91 Log_10_ FFU/well For Makona in tripartite soil (Fig. 1c, Table 1). Residual titer of 3.7 Log_10_ FFU/well is observed for both variants after disinfection in donor blood at 0.02% (Fig. 1c, Table 2).

**Table 1﻿:** Summary of residual infectious titer and Log_10_ fold reduction of Mayinga and Makona after treatment with EtOH, NaOCl, and PAA in tripartite soil. Virucidal conditions are denoted in bold letters and underlined.

	Mayinga			Makona		
	Titer (Log_10_FFU)	± SD	Reduction (Log_10_)	Titer (Log_10_FFU)	± SD	Reduction (Log_10_)
70% EtOH	0.95	0.00	− **4.88**	0.95	0.00	− **5.28**
40% EtOH	3.79	0.28	− 2.04	5.47	0.55	− 0.76
Positive control	5.83	0.26	–	6.23	0.17	–
0.5% NaOCl	0.95	0.00	− **5.03**	0.95	0.00	− **5.30**
0.05% NaOCl	2.20	0.24	− 3.79	1.35	0.08	− **4.91﻿**
Positive control	5.99	0.35	–	6.26	0.15	–
0.2% PAA	0.95	0.00	− **4.85**	0.95	0.00	− **5.25**
0.02% PAA	2.78	0.13	− 3.02	2.91	0.19	− 3.30
Positive control	5.80	0.21	–	6.20	0.12	–

**Table 2﻿:** Summary of residual infectious titer and Log_10_ fold reduction of Mayinga and Makona after treatment with EtOH, NaOCl, and PAA in whole blood. Virucidal conditions are denoted in bold letters and underlined.

	Mayinga			Makona		
	Titer (Log_10_FFU)	± SD	Reduction (Log_10_)	Titer (Log_10_FFU)	± SD	Reduction (Log_10_)
70% EtOH	0.95	0.00	− **4.99**	0.95	0.00	− **5.31**
40% EtOH	1.95	1.00	− **3.99**	1.38	0.43	− **4.88**
Positive control	5.95	0.08	–	6.26	0.07	–
0.5% NaOCl	0.95	0.00	− **4.84**	0.95	0.00	− **5.32**
0.05% NaOCl	5.13	0.26	− 0.67	5.12	0.07	− 1.15
Positive control	5.79	0.22	–	6.28	0.11	–
0.2% PAA	0.95	0.00	− **5.36**	0.95	0.00	− **5.88**
0.02% PAA	3.73	0.11	− 2.58	3.75	0.09	− 3.08
Positive control	6.31	0.19	–	6.83	0.11	–

## Summary of virucidal activity.

**Figure 2:**
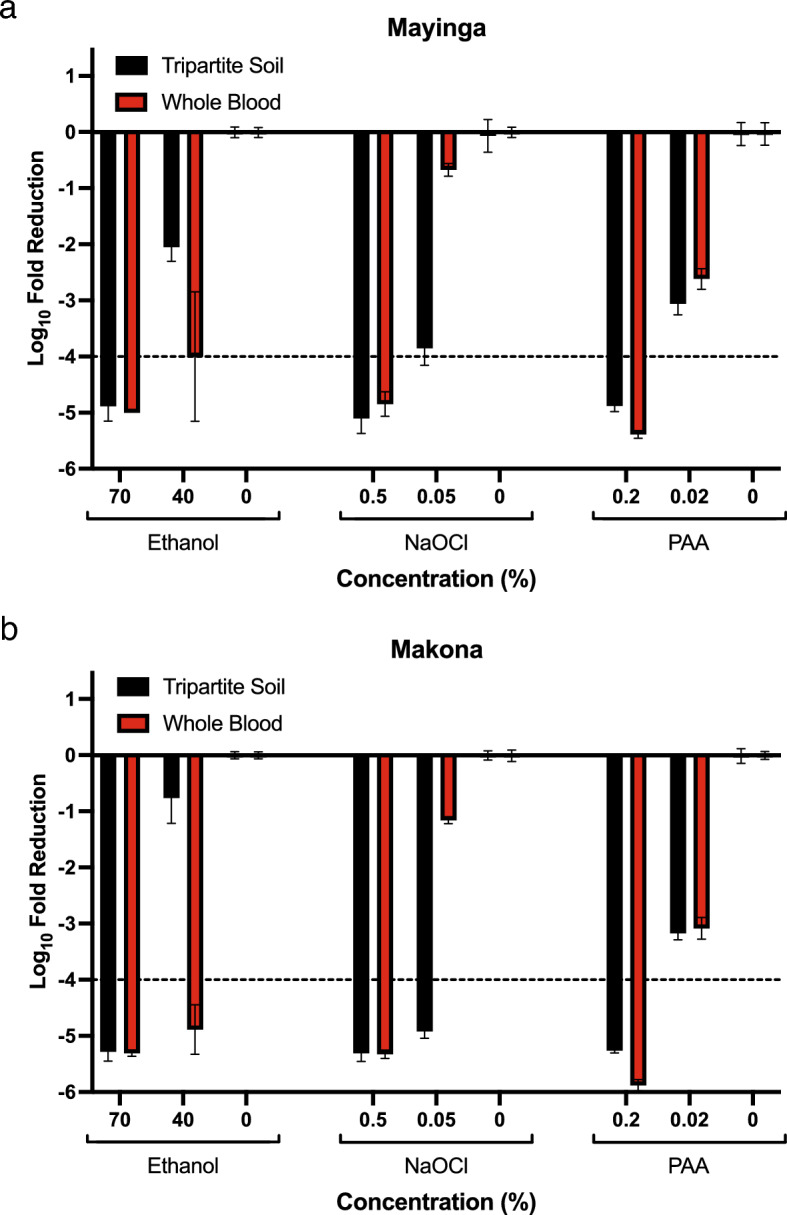
Summary of virucidal activity of three chemical disinfectants against two EBOV strains suspended in two different biological soil types. (**a**) Mayinga and (**b**) Makona. Only minor differences were observed between the two virus variants after treatment with chemical disinfectants at two concentrations for 30 s. Makona appears to be slightly more resistant to 40% ethanol in tripartite soil compared to Mayinga while the opposite is true for 0.05% NaOCl. The pattern of virucidal activity of PAA is the same for both EBOV variants. Corresponding positive controls are indicated as 0%. Dashed line: virucidal activity, ≥ 4 Log_10_ Fold reduction. Bars indicate mean ± SD of 2 independent experiments with n = 6 for test concentrations and n = 4 for controls.

Disinfection of the prototype EBOV variant Mayinga suspended in tripartite soil for 30 s is virucidal (> 4 Log_10_ reduction) as assessed by focus assay for the following conditions: 70% ethanol. 0.5% NaOCl, and 0.2% PAA while the disinfection capacity, presented as Log_10_ reduction, of suboptimal concentrations of disinfectants is 2.04 for 40% ethanol, 3.78 for 0.05% NaOCl, and 3.02 for 0.02% PAA (Fig. 2a and Table 1). When suspended in donor blood, 70% ethanol, 0.5% NaOCl, and 0.2% PAA all retain their virucidal activity against Mayinga. Interestingly, under those conditions the virucidal activity of 40% ethanol increases to borderline virucidal levels (3.99 Log_10_ reduction) and only minor disinfection is observed for 0.05% NaOCl (0.67 Log_10_ reduction) while fold reduction of 2.58 Log_10_ is observed for 0.02% PAA (Fig. 2a, Table 2). Disinfection of the West African outbreak strain﻿, Makona,﻿ suspended in tripartite soil is virucidal for the same conditions observed for Mayinga with the addition of 0.05% NaOCl (4.91 Log_10_ reduction, Fig. 2b, Table 1). In donor blood, both 70 and 40% ethanol are virucidal with 5.31 and 4.88 Log_10_ reduction, respectively. Treatment with 0.5% NaOCl and 0.2% PAA results in over 5 Log_10_ reduction making both conditions virucidal by definition. As observed for Mayinga, the disinfection capacity of 0.05% NaOCl is lost when applied to whole blood compared to tripartite soil (1.15 vs. 4.91 Log_10_ reduction) while 0.02% PAA results in 3.08 Log_10_ reduction after 30 s (Fig. 2b, Table 2). In general,﻿ disinfection﻿ in suboptimal conditions of PAA (0.02%) is less affected by either soil load or virus variant, with observed Log_10_ reduction ranging from 2.58 to 3.30 (Fig. 2, Tables 1 and 2).

## Discussion

Due to the continuous threat of EBOV and other highly virulent zoonotic viruses, reliable chemical disinfection protocols for such viruses are essential for infection prevention and both protocols and chemicals must be regularly tested and validated for efficacy and safety. The protocol described in the current study is intended for in-house testing of chemical disinfectants against highly pathogenic viruses. Standardized testing of disinfectants against viral pathogens is paramount, to ensure the correct assessment of virucidal activity and reproducibility between laboratories. Furthermore, strict experimental controls must be applied to ensure experimental validity. In the current study, all controls were required to be within 0.5 Log of the positive control for each experiment to be considered valid, eliminating experimental uncertainty. In the current study, while developing an in-house testing protocol for disinfectants, we have assessed the suitability and efficacy of 70% ethanol and 0.5% NaOCl as chemical disinfectants against EBOV suspended in either tripartite soil, a substitute for mucosal secretions, or donor blood. These chemical disinfectants are either suggested for the general inactivation of enveloped viruses (70% ethanol) or directly recommended by WHO for EBOV disinfection (0.5% NaOCl)^18–21^. Furthermore, we have assessed the suitability of PAA as a potential chemical disinfectant against EBOV and found it to be virucidal at a low concentration (0.2%) and less affected by the different soil loads compared to ethanol and NaOCl. PAA is becoming increasingly popular as a disinfectant in laboratory settings, especially in high containment laboratories in Europe^22–24^. A potential benefit of PAA usage in the field would be an overall lower end-concentration of disinfectant, as we observed 0.2% to be virucidal, and the abundance and availability of PAA due to its frequent use in the nutritional industry^25–27^. This could be beneficial for disinfection in remote locations with unstable supply chains and infrastructure. Additionally, PAA breaks down to water and acetic acid hence the environmental impact of PAA waste after disinfection would be less harmful compared to NaOCl in field situations where waste cannot be disposed of in a controlled manner. However, the accessibility of PAA in rural Africa could present a potential downside of PAA usage during viral epidemics since bleach (NaOCl) is widely available as a stock solution or already incorporated into household cleaning products and can be used in epidemic situations safely and swiftly. Additionally, the limited storage conditions for liquid PAA (15–25 °C, protected from sunlight, good ventilation) might prove challenging for direct field usage. Therefore, the use of PAA in epidemic situations might be better suited for more controlled indoor environments. Based on the results obtained in the current study, we would suggest that the choice of a chemical disinfectant should primarily depend on the biological soil load and special care must be taken to ensure the proper end-concentration for complete inactivation of infectious virus, especially when dealing with larger volumes and ready-to-use disinfectants, e.g., pre-diluted 70% ethanol for laboratories and hospitals. Indeed, our data showed that suboptimal concentrations of the tested disinfectants failed to completely inactivate both ZEBOV variants while the type of biological soil differentially impacted the efficacy of the disinfection, especially when treated with ethanol and NaOCl. Additionally, some differences between the two virus variants were also observed upon treatment with suboptimal concentrations. In general, ≥ 4 Log_10_ reduction is traditionally considered virucidal for chemical disinfectants, as it corresponds to 99.99% reduction of viral titer^28,29^. However we must point out that incomplete inactivation of highly pathogenic and infectious viruses during outbreaks might have devastating outcomes. This means that higher Log_10_ reductions should be anticipated (i.e., complete inactivation of infectious virus) for evaluation of chemical disinfectants against such viruses. To assess the overall suitability of PAA as a general disinfectant for highly pathogenic viruses, future research should aim to comprehensively define its inactivation properties using infectious human material and determine its suitability for disinfection of contaminated complex surfaces.

### Supplementary Information

Supplementary Information.

## Data Availability

All source data are available from the corresponding authors. H.R.J. or D.Z. upon reasonable request.

## References

[1] Goeijenbier M, van Kampen JJ, Reusken CB, Koopmans MP, van Gorp EC (2014). Ebola virus disease: A review on epidemiology, symptoms, treatment and pathogenesis. Neth J Med.

[2] Guan J, Chan M, Brooks BW, Rohonczy L (2013). Influence of temperature and organic load on chemical disinfection of Geobacillus steareothermophilus spores, a surrogate for *Bacillus anthracis*. Can. J. Vet. Res..

[3] Cook BWM (2016). The disinfection characteristics of ebola virus outbreak variants. Sci. Rep..

[4] Rossi S, Antonelli M, Mezzanotte V, Nurizzo C (2007). Peracetic acid disinfection: A feasible alternative to wastewater chlorination. Water Environ. Res..

[5] Cutts T, Kasloff S, Safronetz D, Krishnan J (2021). Decontamination of common healthcare facility surfaces contaminated with SARS-CoV-2 using peracetic acid dry fogging. J. Hosp. Infect..

[6] Kruszewska E (2021). Fogging with peracetic acid in schools and kindergartens. Front. Public Heal..

[7] Kruszewska E (2022). Is peracetic acid fumigation effective in public transportation?. Int. J. Environ. Res. Public Heal..

[8] Günther S (2011). Management of accidental exposure to Ebola virus in the biosafety level 4 laboratory, Hamburg, Germany. J. Infect. Dis..

[9] Luukkonen T, Teeriniemi J, Prokkola H, Rämö J, Lassi U (2014). Chemical aspects of peracetic acid based wastewater disinfection. Water SA.

[10] Cavallini GS, De Campos SX, De Souza JB, Vidal CMS (2013). Evaluation of the physical-chemical characteristics of wastewater after disinfection with peracetic acid. Water Air Soil Pollut..

[11] Megahed A, Aldridge B, Lowe J (2019). Comparative study on the efficacy of sodium hypochlorite, aqueous ozone, and peracetic acid in the elimination of Salmonella from cattle manure contaminated various surfaces supported by Bayesian analysis. PLoS ONE.

[12] Rabenau HF (2015). Guideline of the German association for the control of viral diseases (DVV) eV and the Robert Koch Institute (RKI) for testing chemical disinfectants for effectiveness against viruses in human medicine. Version of 1 December, 2014. Bundesgesundheitsblatt Gesundheitsforschung Gesundheitsschutz.

[13] Cutts TA (2020). Efficacy of microbicides for inactivation of Ebola-Makona virus on a non-porous surface: a targeted hygiene intervention for reducing virus spread. Sci. Rep..

[14] Springthorpe VS, Sattar SA (2005). Carrier tests to assess microbicidal activities of chemical disinfectants for use on medical devices and environmental surfaces. J. AOAC Int..

[15] Cook BWM (2015). Evaluating environmental persistence and disinfection of the Ebola Virus Makona variant. Viruses.

[16] Ackermann-Gäumann R (2019). Standardized focus assay protocol for biosafety level four viruses. J. Virol. Methods.

[17] Trombley AR (2010). Comprehensive panel of real-time TaqMan polymerase chain reaction assays for detection and absolute quantification of filoviruses, arenaviruses, and New World hantaviruses. Am. J. Trop. Med. Hyg..

[18] Sauerbrei A (2020). Bactericidal and virucidal activity of ethanol and povidone-iodine. MicrobiologyOpen.

[19] Kampf G (2018). Efficacy of ethanol against viruses in hand disinfection. J. Hosp. Infect..

[20] Nomura T (2021). Ethanol susceptibility of SARS-CoV-2 and other enveloped viruses. Biocontrol Sci..

[21] World Health Organization, Clinical management of patients with viral haemorrhagic fever: A pocket guide for front-line health workers: Interim emergency guidance for country adaptation, https://apps.who.int/iris/handle/10665/205570 (2016).

[22] Rybka A (2021). Peracetic acid-based disinfectant is the most appropriate solution for a biological decontamination procedure of responders and healthcare workers in the field environment. J. Appl. Microbiol..

[23] Loibner M, Langner C, Regitnig P, Gorkiewicz G, Zatloukal K (2021). Biosafety requirements for autopsies of patients with COVID-19: Example of a BSL-3 autopsy facility designed for highly pathogenic agents. Pathobiology.

[24] Kuemin D, Krebs C, Wick P (2011). How to choose a suit for a BSL-4 laboratory-the approach taken at SPIEZ laboratory. Appl. Biosaf..

[25] Zoellner C, Aguayo-Acosta A, Siddiqui MW, Dávila-Aviña JE (2018). Postharvest disinfection of fruits and vegetables. Postharvest disinfection of fruits and vegetables.

[26] Nicolau-Lapeña I (2019). Strawberry sanitization by peracetic acid washing and its effect on fruit quality. Food Microbiol..

[27] Scaramuzza N, Mutti P, Cigarini M, Berni E (2020). Effect of peracetic acid on ascospore-forming molds and test microorganisms used for bio-validations of sanitizing processes in food plants. Int. J. Food Microbiol..

[28] Steinhauer K (2021). Virucidal efficacy of different formulations for hand and surface disinfection targeting SARS CoV-2. J. Hosp. Infect..

[29] Tarka P, Nitsch-Osuch A (2021). Evaluating the virucidal activity of disinfectants according to European Union standards. Viruses.

