# Supplementary material for: Virucidal activity of three standard chemical disinfectants against Ebola virus suspended in tripartite soil and whole blood
Source: Sci Rep. 2023 Sep 21;13:15718. doi: 10.1038/s41598-023-42376-8 (PMC10514052; doi:10.1038/s41598-023-42376-8)
Supplement: Supplementary file 1 — Supplementary Information. [file 41598_2023_42376_MOESM1_ESM.pdf]

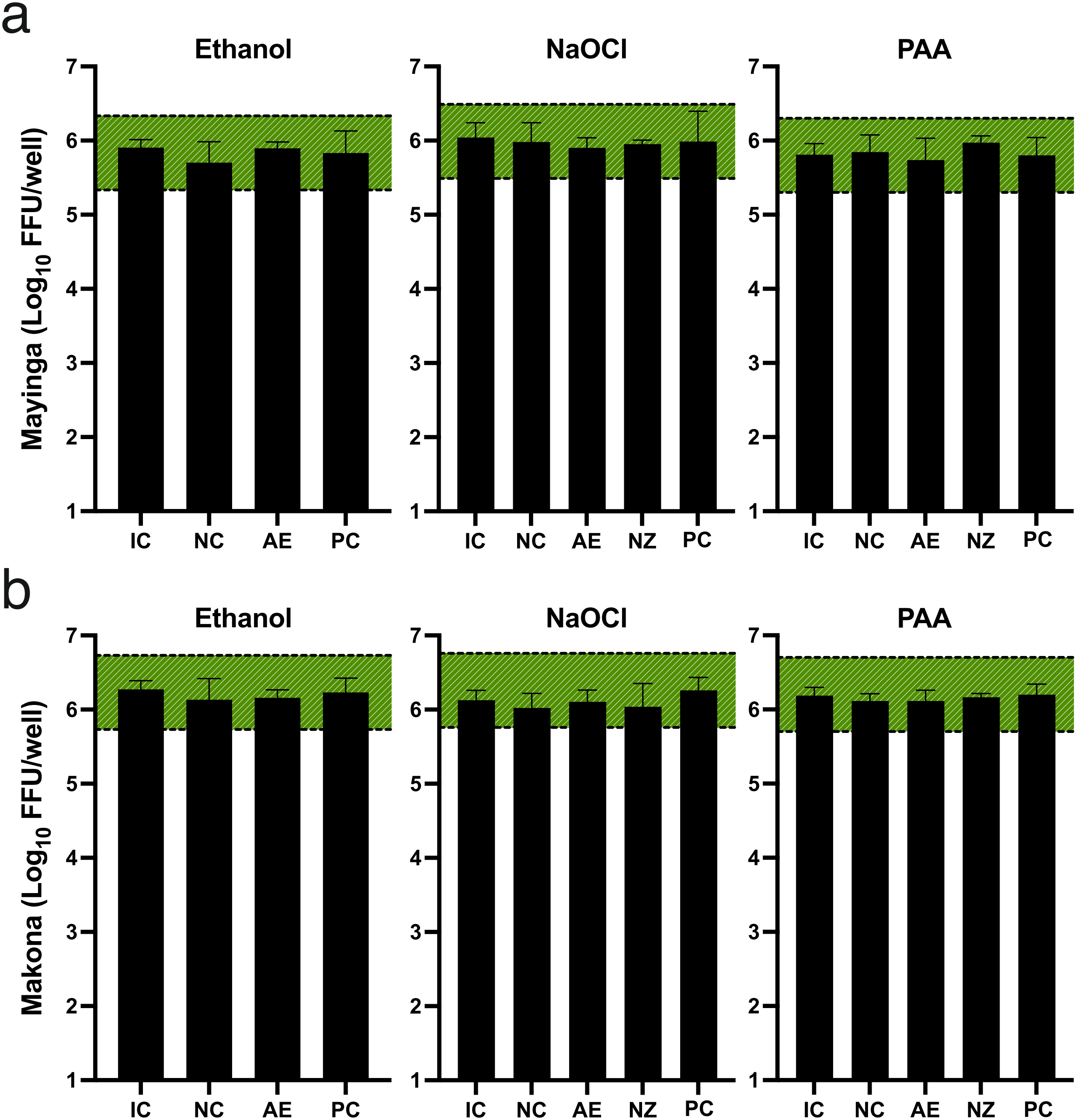
a
Ethanol
NaOCl
PAA
Mayinga (Log10 FFU/well)
IC
NC
AE
NZ
PC
b
Ethanol
NaOCl
PAA
Makona (Log10 FFU/well)
IC
NC
AE
NZ
PC

**Figure S1: Overview of controls.** Internal controls applied to assess experimental validity for inactivation of EBOV Mayinga in a) tripartite soil and b) donor blood. IC: interference control disinfectant, NC: interference control PBS, AE: after-effects control, NZ: neutralizer control, PC: positive control. Dashed green: zone of experimental validity compared to PC. Bars indicate mean ±SD of 2 independent experiments with n=4.

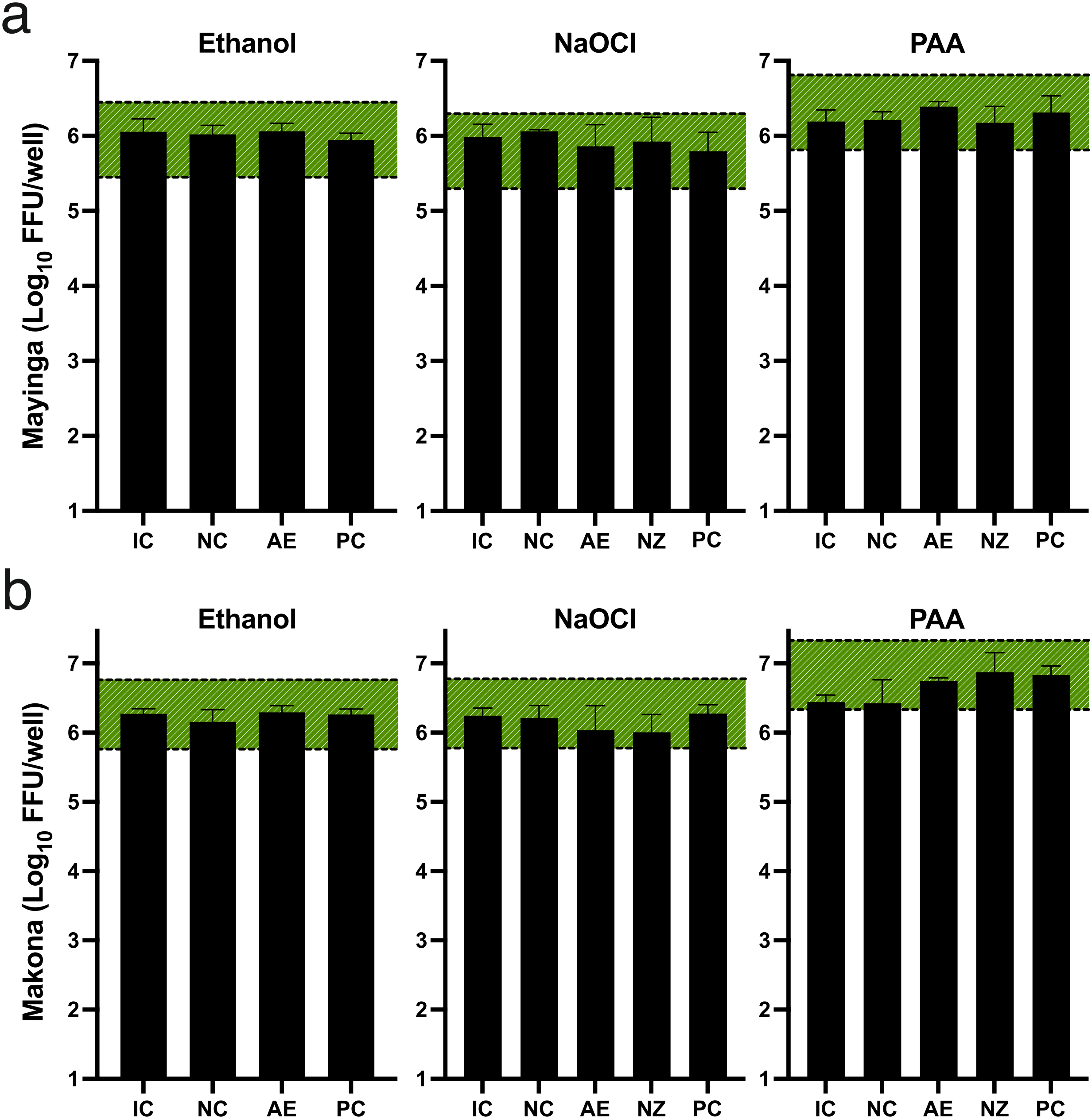
a
Ethanol
NaOCl
PAA
Mayinga (Log10 FFU/well)
IC
NC
AE
NZ
PC
b
Makona (Log10 FFU/well)
1
2
3
4
5
6
7

**Figure S2: Overview of controls.** Internal controls applied to assess experimental validity for inactivation of EBOV Makona in a) tripartite soil and b) donor blood. IC: interference control disinfectant, NC: interference control PBS, AE: after-effects control, NZ: neutralizer control, PC: positive control. Dashed green: zone of experimental validity compared to PC. Bars indicate mean ±SD of 2 independent experiments with n=4.

| | Mayinga | | | Makona | | |
|---|---|---|---|---|---|---|
| | Titer ($Log_{10}$FFU) | ±SD | Reduction ($Log_{10}$) | Titer ($Log_{10}$FFU) | ±SD | Reduction ($Log_{10}$) |
| **70% EtOH** | 0,95 | 0,00 | **-4,88** | 0,95 | 0,00 | **-5,28** |
| **40% EtOH** | 3,79 | 0,28 | -2,04 | 5,47 | 0,55 | -0,76 |
| **Interference EtOH** | 5,91 | 0,09 | 0,07 | 6,27 | 0,10 | 0,04 |
| **Interference PBS** | 5,70 | 0,24 | -0,13 | 6,13 | 0,25 | -0,10 |
| **After Effects control** | 5,90 | 0,07 | 0,06 | 6,16 | 0,09 | -0,07 |
| **Virus control** | 5,83 | 0,26 | 0,00 | 6,23 | 0,17 | - |
| | | | | | | |
| **0.5% NaOCl** | 0,95 | 0,00 | **-5,03** | 0,95 | 0,00 | **-5,30** |
| **0.05% NaOCl** | 2,20 | 0,24 | -3,78 | 1,35 | 0,08 | **-4,91** |
| **Interference NaOCl** | 6,04 | 0,17 | 0,05 | 6,12 | 0,12 | -0,14 |
| **Interference PBS** | 5,98 | 0,22 | -0,01 | 6,02 | 0,17 | -0,24 |
| **After Effects control** | 5,90 | 0,12 | -0,09 | 6,10 | 0,14 | -0,16 |
| **Neutralizer control** | 5,95 | 0,05 | -0,04 | 6,03 | 0,27 | -0,22 |
| **Virus control** | 5,99 | 0,35 | 0,00 | 6,26 | 0,15 | - |
| | | | | | | |
| **0.2% PAA** | 0,95 | 0,00 | **-4,85** | 0,95 | 0,00 | **-5,25** |
| **0.02% PAA** | 2,78 | 0,13 | -3,02 | 2,91 | 0,19 | -3,30 |
| **Interference PAA** | 5,81 | 0,13 | 0,01 | 6,19 | 0,10 | -0,02 |
| **Interference PBS** | 5,85 | 0,20 | 0,05 | 6,11 | 0,08 | -0,09 |
| **After effects control** | 5,74 | 0,26 | -0,06 | 6,11 | 0,13 | -0,09 |
| **Neutralizer control** | 5,97 | 0,08 | 0,17 | 6,17 | 0,04 | -0,03 |
| **Virus control** | 5,80 | 0,21 | 0,00 | 6,20 | 0,12 | - |

**Table S1: Summary of the $Log_{10}$ fold reduction of Mayinga and Makona after treatment with EtOH, NaOCl, PAA, and all internal controls in tripartite soil**. Virucidal conditions are denoted in bold letters and underlined.

| | Mayinga | | | Makona | | |
|---|---|---|---|---|---|---|
| | Titer ($Log_{10}$FFU) | ±SD | Reduction ($Log_{10}$) | Titer ($Log_{10}$FFU) | ±SD | Reduction ($Log_{10}$) |
| **70% EtOH** | 0,95 | 0,00 | **-4,99** | 0,95 | 0,00 | **-5,31** |
| **40% EtOH** | 1,95 | 1,00 | -3,99 | 1,38 | 0,43 | **-4,88** |
| **Interference EtOH** | 6,06 | 0,15 | 0,11 | 6,27 | 0,06 | 0,01 |
| **Interference PBS** | 6,02 | 0,10 | 0,07 | 6,16 | 0,15 | -0,10 |
| **After Effects control** | 6,06 | 0,09 | 0,11 | 6,29 | 0,08 | 0,03 |
| **Virus control** | 5,95 | 0,08 | 0,00 | 6,26 | 0,07 | 0,00 |
| | | | | | | |
| **0.5% NaOCl** | 0,95 | 0,00 | **-4,84** | 0,95 | 0,00 | **-5,32** |
| **0.05% NaOCl** | 5,13 | 0,26 | -0,67 | 5,12 | 0,07 | -1,15 |
| **Interference NaOCl** | 5,99 | 0,15 | 0,19 | 6,24 | 0,09 | -0,03 |
| **Interference PBS** | 6,06 | 0,02 | 0,27 | 6,21 | 0,16 | -0,06 |
| **After Effects control** | 5,86 | 0,25 | 0,07 | 6,04 | 0,30 | -0,24 |
| **Neutralizer control** | 5,92 | 0,28 | 0,13 | 6,01 | 0,22 | -0,27 |
| **Virus control** | 5,79 | 0,22 | 0,00 | 6,28 | 0,11 | 0,00 |
| | | | | | | |
| **0.2% PAA** | 0,95 | 0,00 | **-5,36** | 0,95 | 0,00 | **-5,88** |
| **0.02% PAA** | 3,73 | 0,11 | -2,58 | 3,75 | 0,09 | -3,08 |
| **Interference PAA** | 6,19 | 0,13 | -0,12 | 6,44 | 0,09 | -0,39 |
| **Interference PBS** | 6,21 | 0,09 | -0,10 | 6,42 | 0,29 | -0,41 |
| **After effects control** | 6,39 | 0,06 | 0,08 | 6,74 | 0,04 | -0,09 |
| **Neutralizer control** | 6,17 | 0,19 | -0,14 | 6,87 | 0,25 | 0,04 |
| **Virus control** | 6,31 | 0,19 | 0,00 | 6,83 | 0,11 | 0,00 |

**Table S2: Summary of the $Log_{10}$ fold reduction of Mayinga and Makona after treatment with EtOH, NaOCl, PAA, and all internal controls in whole blood**. Virucidal conditions are denoted in bold letters and underlined.

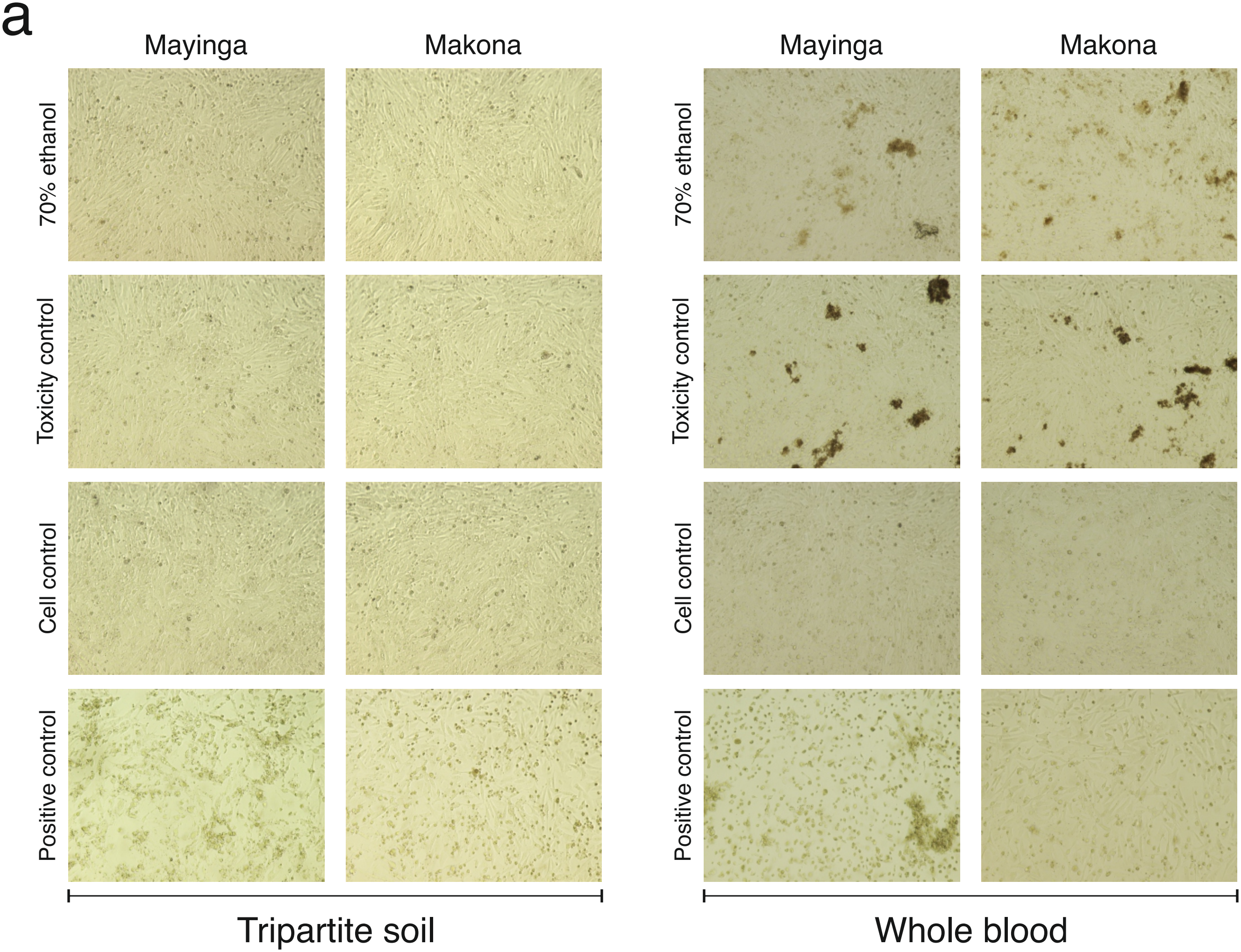
a
Mayinga
Makona
Mayinga
Makona
70% ethanol
Toxicity control
Cell control
Positive control
70% ethanol
Toxicity control
Cell control
Positive control
Tripartite soil
Whole blood

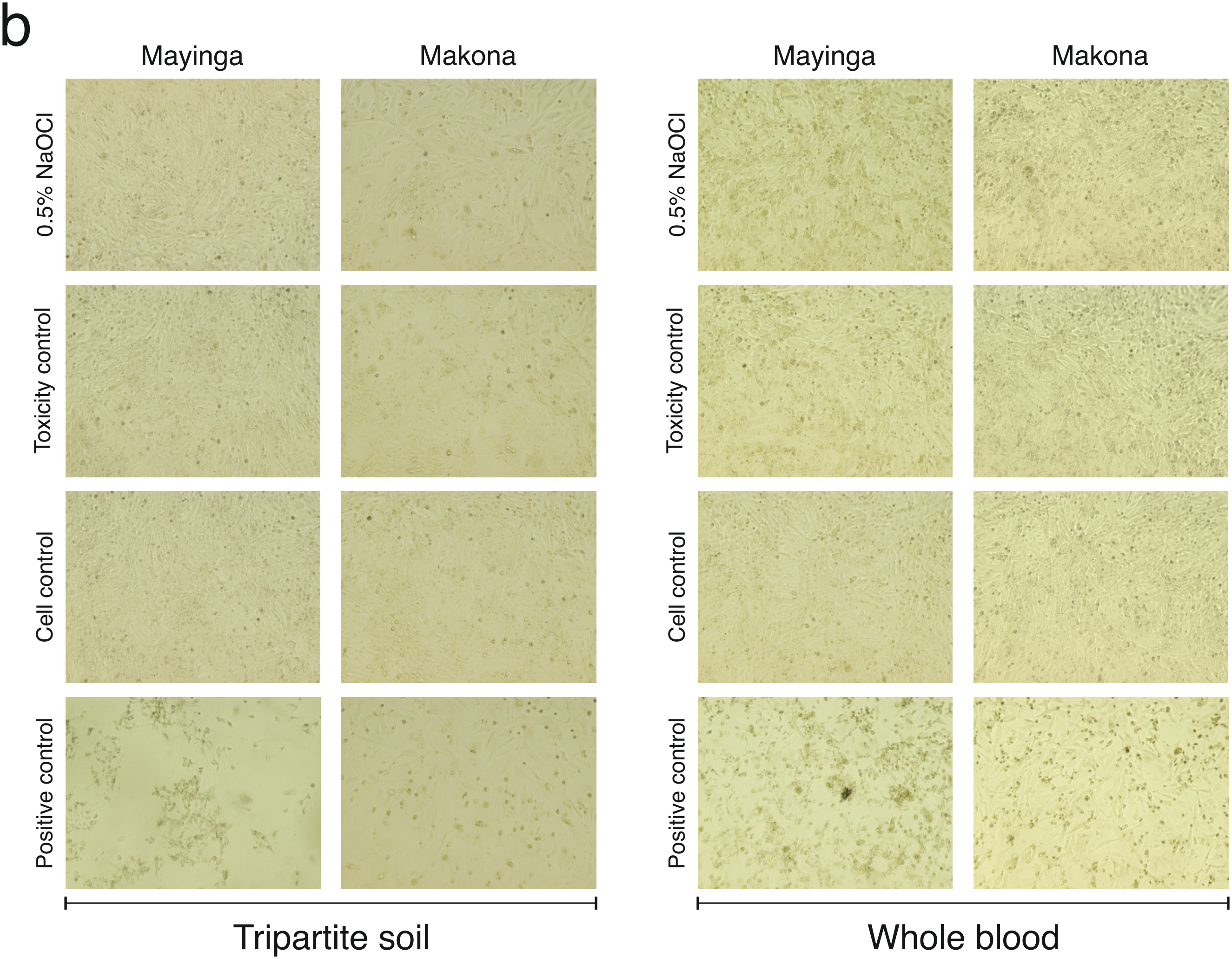
b
Mayinga
Makona
Mayinga
Makona
0.5% NaOCl
Toxicity control
Cell control
Positive control
0.5% NaOCl
Toxicity control
Cell control
Positive control
Tripartite soil
Whole blood

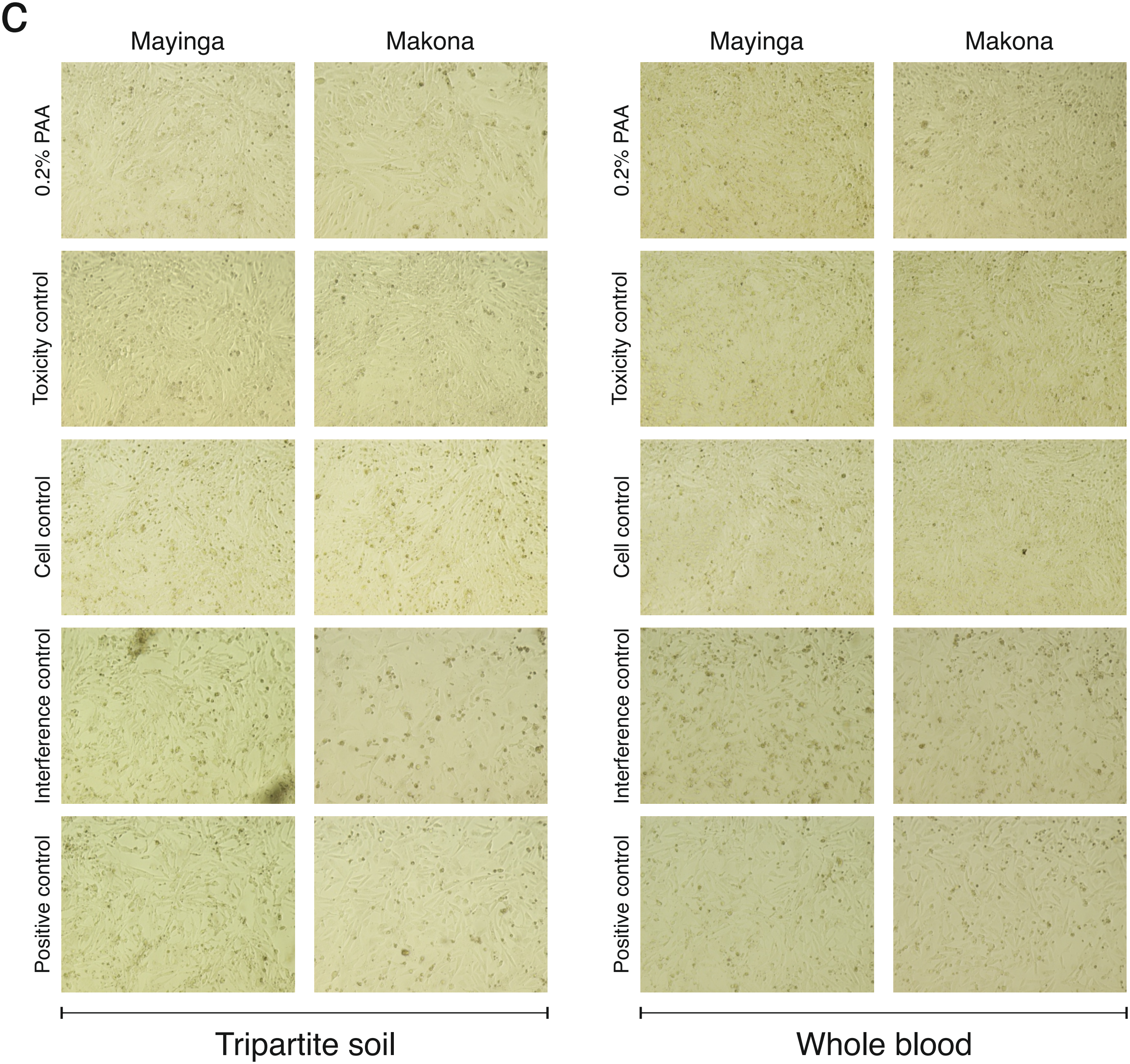
C
Mayinga
Makona
Mayinga
Makona
0.2% PAA
Toxicity control
Cell control
Interference control
Positive control
0.2% PAA
Toxicity control
Cell control
Interference control
Positive control
Tripartite soil
Whole blood

**Figure S3: Representative examples of CPE assessment in complete kill controls by microscopy.** No CPE is observed in complete kill controls after 7- day incubation with EBOV treated with a) 70 % ethanol, b) 0.5 % NaOCl, and c) 0.2 % PAA for 30 s. CPE is observed only in positive controls, as expected, and interference controls for PAA. Original magnification: 10x.

| | Tripartite Soil | | | | Blood | | |
|---|---|---|---|---|---|---|---|
| | | | | | | | |
| | d0 | d7 | ΔCt (d0-d7) | | d0 | d7 | ΔCt (d0-d7) |
| **70% EtOH** | 28.28 | 29.38 | -1.10 | | 30.025 | 32.51 | -2.485 |
| | 28.82 | 29.54 | -0.73 | | 29.345 | 31.5 | -2.155 |
| | 29.00 | 30.19 | -1.19 | | 32.055 | 35.215 | -3.16 |
| **Positive control** | 27.22 | 19.22 | 8.00 | | 27.275 | 18.775 | 8.5 |
| | | | | | | | |
| | d0 | d7 | ΔCt (d0-d7) | | d0 | d7 | ΔCt (d0-d7) |
| **0.5% NaOCl** | 34.115 | 33.425 | 0.69 | | 29.425 | 30.4 | -0.975 |
| | 33.86 | 34.84 | -0.98 | | 28.895 | 30.055 | -1.16 |
| | 36.285 | 35.08 | 1.205 | | 29.42 | 29.65 | -0.23 |
| **Positive control** | 29.7 | 18.59 | 11.11 | | 27.705 | 18.87 | 8.835 |
| | | | | | | | |
| | d0 | d7 | ΔCt (d0-d7) | | d0 | d7 | ΔCt (d0-d7) |
| **0.2% PAA** | 27.045 | 27.745 | -0.7 | | 27.105 | 28.75 | -1.645 |
| | 27.8825 | 27.8225 | 0.06 | | 27.37 | 28.9075 | -1.5375 |
| | 27.1925 | 27.86 | -0.6675 | | 27.3825 | 29.5 | -2.1175 |
| **Interference control** | 27.0525 | 20.235 | 6.8175 | | 26.925 | 19.8775 | 7.0475 |
| **Positive control** | 26.8 | 19.79 | 7.01 | | 27.2325 | 20.455 | 6.7775 |

**Table S3a: Summary of EBOV Mayinga replication in complete kill controls.** Inactivation of EBOV was assessed by qRT-PCR in complete kill control supernatant. Delta Ct values ≥ 3 were considered indicative of active replication.

| | Tripartite Soil | | | | Blood | | |
|---|---|---|---|---|---|---|---|
| | | | | | | | |
| | d0 | d7 | ΔCt (d0-d7) | | d0 | d7 | ΔCt (d0-d7) |
| **70% EtOH** | 26.85 | 27.60 | -0.75 | | 27.685 | 30.32 | -2.635 |
| | 26.67 | 26.95 | -0.28 | | 29.23 | 32.52 | -3.29 |
| | 27.56 | 28.03 | -0.47 | | 29.345 | 32 | -2.655 |
| **Positive control** | 26.41 | 17.53 | 8.88 | | 27.43 | 17.74 | 9.69 |
| | | | | | | | |
| | d0 | d7 | ΔCt (d0-d7) | | d0 | d7 | ΔCt (d0-d7) |
| **0.5% NaOCl** | 34.055 | 35.375 | -1.32 | | 29.875 | 30.34 | -0.465 |
| | 35.12 | 34.62 | 0.5 | | 30.235 | 30.73 | -0.495 |
| | 36.99 | 35.005 | 1.985 | | 30.015 | 29.995 | 0.02 |
| **Positive control** | 26.85 | 19.46 | 7.39 | | 26.945 | 17.5 | 9.445 |
| | | | | | | | |
| | d0 | d7 | ΔCt (d0-d7) | | d0 | d7 | ΔCt (d0-d7) |
| **0.2% PAA** | 25.99 | 26.4875 | -0.4975 | | 26.3075 | 27.99 | -1.6825 |
| | 25.9675 | 26.64 | -0.6725 | | 26.1625 | 27.1 | -0.9375 |
| | 26.1975 | 26.63 | -0.4325 | | 27.35 | 27.0325 | 0.3175 |
| **Interference control** | 26.3125 | 18.065 | 8.2475 | | 26.92 | 17.045 | 9.875 |
| **Positive control** | 26.055 | 17.655 | 8.4 | | 26.51 | 17.01 | 9.5 |

**Table S3b: Summary of EBOV Makona replication in complete kill controls.** Inactivation of EBOV was assessed by qRT-PCR in complete kill control supernatant. Delta Ct values ≥ 3 were considered indicative of active replication.
